# Supplementary material for: The role of online social networks in improving health literacy and medication adherence among people living with HIV/AIDS in Iran: Development of a conceptual model
Source: PLoS One. 2022 Jun 30;17(6):e0261304. doi: 10.1371/journal.pone.0261304 (PMC9246123; doi:10.1371/journal.pone.0261304)
Supplement: S1 Appendix — (DOCX) [file pone.0261304.s001.docx]

| **Document** | **Code** | **Segment** |
| --- | --- | --- |
| Interview 1 | Sex\female | Female |
| Interview 1 | age | 46 years old |
| Interview 1 | education | I have diploma |
| Interview 1 | employment status | Now neither I nor my husband is employed ... we are both unemployed |
| Interview 1 | way of transmission | I had surgery a few years ago and they injected me with blood |
| Interview 1 | date of diagnosis | I have been under treatment since [Date] |
| Interview 1 | history of drug abuse | How about a history of drug use? Not at all |
| Interview 1 | OSN app | I only use Telegram |
| Interview 1 | Communication support\Patient-provider | The online group that the colleagues set up is very good for communicating with specialist doctors, and you can leave a message and receive a response at any time. |
| Interview 1 | Communication support\Patient-provider | Psychologists are members of the online groups |
| Interview 1 | OSN group | Shoma Ba Ma |
| Interview 1 | OSN group | Yaran-e-Mosbat |
| Interview 1 | Time of using OSN | not more than an hour |
| Interview 1 | Communication support | To interact with friends, and get in touch |
| Interview 1 | Communication support\Peer communication | Get in touch with other patients |
| Interview 1 | Knowledge about HIV | To become aware of the HIV and its complications |
| Interview 1 | Communication support | The groups are extremely valuable and excellent…we have been friends for over …years. We usually discuss and share our problems and personal issues with friends and try to find a solution by collaboration. I usually persuade my friends to join training courses about HIV and interact with other patients regardless of gender issues. |
| Interview 1 | Communication support | Because of these advantages, we have made it more possible for patients to be in close contact with each other, to be able to share their issues with each other, to communicate, and raise their issues. |
| Interview 1 | Communication support\Peer communication | Patients have a close relationship with each other |
| Interview 1 | Emotional support | Do you feel close to each other? Too much |
| Interview 1 | Informational support | Our disease is such that some issues that arise for patients cannot be said in public, for example, a young woman had genital warts and several other health problems that made her extremely worried and anxious. Her mother contacted me to seek my advice. I helped her visit a doctor and find information sources for her queries. |
| Interview 1 | Access to information | When we have a problem, we usually do not have access to the doctor on time. By using online social networks, we have the opportunity to leave a message and the physician answers our questions 24/7. This is a great advantage for patients that usually do not use health services and rarely visit a doctor due to social stigma. |
| Interview 1 | Knowledge about HIV | I have learned a lot of new things since I came here. We usually talk about our illness. In addition to learning a lot of new things about our illness, many things are added to our information. |
| Interview 1 | Self-efficacy & self-care behavior | Exercising, walking, swimming, going to the theater regularly, and the cinema are my routine life. I am a stable person, and I advise my children and friends to be like me. |
| Interview 1 | Motivation & Confidence | Listening to our friends’ problems and empathizing with them let them know that we have felt the same way too.  When we do this, we let our friends know that we love them. This motivates her to continue with a difficult situation and survive. |
| Interview 1 | Emotional support | Believe that suspending online social networks will hurt the patients. Access to mobile internet packages is somehow cheap for patients and they can afford the financial issues. By using online social networks, they are allowed to communicate with other patients, share their feelings and experiences, and receive emotional support from the community. |
| Interview 1 | Knowledge about HIV | Online social networks really work for patients as they can access to reliable and updated health information that is provided by physicians and health personnel through these applications. Finding information about drug adverse effects, complementary therapies, herbal medicine, and nutrition are exciting topics that are routinely discussed among the patients and the online discussions help the patient find the best alternative for their treatment. |
| Interview 1 | Disease perception | Some people think that HIV is transmitted by kissing, saliva, or eating water while swimming in a public pool. |
| Interview 1 | Motivation & Confidence | By observing patients that are well coping with the disease, patients are motivated and learn how to manage their illness. |
| Interview 1 | Recommendations for OSN improvements | Online social networks help communicate with communities that are not easily accessible. By establishing a virtual community of youth, vulnerable populations, and HIV-positive volunteers, we can convince guys to take protective strategies such as using condoms to prevent acquiring HIV from their partners. |
| Interview 1 | Disease perception | I’d always thought that HIV is a killing disease. |
| Interview 1 | Communication support\trust | Most information about HIV is shared via online social networks and the internet is not reliable and correct. For example, recently a fake news was distributed among the patients about the certain drug for HIV. This caused a group of patients to interrupt or postpone their antiretroviral treatment regimens. In these situations, online social networks that provide reliable health information are valued and increase trust among patients and healthcare providers. |
| Interview 1 | Emotional support | Communicating with other patients causes good feelings and a sense of motivation. |
| Interview 1 | Knowledge about HIV | Online social networks really improve the users’ knowledge about HIV. |
| Interview 1 | Access to information | Due to social harms experienced by most patients, they usually avoid using health services and visiting physicians. However, using online social networks help them overcome the disadvantages of social stigma and share their problems and questions with a trusted and reliable health expert, and access valuable health information. |
| Interview 1 | Communication support | If experienced physicians join the virtual community and communicate with patients through online initiatives, patients will be increasingly attracted to join the groups and use the services provided online. |
| Interview 1 | Adherence | Online communication with other patients will definitely improve medication adherence and treatment outcomes among the HIV-positive community. |
