# Supplementary material for: The role of online social networks in improving health literacy and medication adherence among people living with HIV/AIDS in Iran: Development of a conceptual model
Source: PLoS One. 2022 Jun 30;17(6):e0261304. doi: 10.1371/journal.pone.0261304 (PMC9246123; doi:10.1371/journal.pone.0261304)
Supplement: S2 Appendix — (DOCX) [file pone.0261304.s002.docx]

**Code System [948]**

**anonymous identity [3]**

**age [28]**

**education [27]**

**history of drug abuse [25]**

**employment status [28]**

**date of diagnosis [29]**

**Sex [0]**

**male [18]**

**female [11]**

**way of transmission [28]**

**Disease perception [16]**

**Knowledge about HIV [45]**

**Access to information [16]**

**Emotional support [53]**

**Informational support [67]**

**Negative emotions [2]**

**Instrumental support [10]**

**Barriers [58]**

**OSN app [39]**

**OSN group [31]**

**Recommendations for OSN improvements [45]**

**Time of using OSN [25]**

**Overall impression [70]**

**Sef efficacy & self-care behavior [19]**

**Adherence [33]**

**Motivation & confidence [56]**

**OSN outcome [0]**

**Overall OSN Use & Impression [2]**

**Communication support [53]**

**Peer communication [33]**

**Patient-provider [26]**

**trust [52]**

**Demographic information [0]**

**Sets [0]**
