# Supplementary material for: The role of online social networks in improving health literacy and medication adherence among people living with HIV/AIDS in Iran: Development of a conceptual model
Source: PLoS One. 2022 Jun 30;17(6):e0261304. doi: 10.1371/journal.pone.0261304 (PMC9246123; doi:10.1371/journal.pone.0261304)
Supplement: S1 File — (HTM) [file pone.0261304.s003.htm]

|  |  |  |  |  |  |  |  |  |  |  |
| --- | --- | --- | --- | --- | --- | --- | --- | --- | --- | --- |
| **Coded segments** | | | | | | | | | | |
| **Comment** | **Document** | **Code** | **Begin** | **End** | **Weight score** | **Segment** | **Author** | **Creation date** | **Document group** | **Page** |
|  | Interview 1 | Sex\female | 3 | 3 | 0 | ز | bazrafshan | 16/11/2019 12:41 |  | 1 |
|  | Interview 1 | age | 4 | 4 | 0 | من 46 سالمه الا | bazrafshan | 16/11/2019 12:41 |  | 1 |
|  | Interview 1 | education | 5 | 5 | 0 | من دیپلم هست | bazrafshan | 16/11/2019 12:41 |  | 1 |
|  | Interview 1 | employment status | 6 | 6 | 0 | الان نه من شاغل هستم نه همسرم ..هر دو بیکاریم | bazrafshan | 16/11/2019 12:42 |  | 1 |
|  | Interview 1 | way of transmission | 8 | 8 | 0 | من سال ... خون گرفتم ی جراحی داشتم | bazrafshan | 16/11/2019 12:43 |  | 1 |
|  | Interview 1 | date of diagnosis | 9 | 9 | 0 | .از اردیبهشت ... تحت درمانم تا الا | bazrafshan | 16/11/2019 12:44 |  | 1 |
|  | Interview 1 | history of drug abuse | 11 | 11 | 0 | سابقه مصرف مواد مخدر چطور؟ اصلاً به هیچ عنوا | bazrafshan | 16/11/2019 12:44 |  | 1 |
|  | Interview 1 | OSN app | 15 | 15 | 0 | من فقط از تلگرام استفاده می کنم | bazrafshan | 16/11/2019 12:45 |  | 1 |
|  | Interview 1 | Communication support\Patient-provider | 16 | 16 | 0 | که بچه های خودمون با پزشکان متخصص گذاشتند که پرسش و پاسخه تایم خاصی ندار | bazrafshan | 19/11/2019 11:45 |  | 1 |
|  | Interview 1 | Communication support\Patient-provider | 16 | 16 | 0 | پزشکان روان شناس ادد هستند تو گوشی | bazrafshan | 19/11/2019 11:46 |  | 1 |
|  | Interview 1 | OSN group | 16 | 16 | 0 | شما با ماس | bazrafshan | 16/11/2019 12:46 |  | 1 |
|  | Interview 1 | OSN group | 16 | 16 | 0 | یاران مثب | bazrafshan | 16/11/2019 12:47 |  | 1 |
|  | Interview 1 | Time of using OSN | 17 | 17 | 0 | نهایت یک ساعت اما نه به طور مداوم معمولا تایمی را میگذارم میخونم بعد مجددا دوباره از یک ساعت یک ساعت و نیم بیشتر نیس | bazrafshan | 16/11/2019 12:46 |  | 1 |
|  | Interview 1 | Communication support | 18 | 18 | 0 | تعامل با دوستان، ارتباط گرفت | bazrafshan | 16/11/2019 12:49 |  | 1 |
|  | Interview 1 | Communication support\Peer communication | 18 | 18 | 0 | تعامل با دوستان، ارتباط گرفت | bazrafshan | 19/11/2019 11:43 |  | 1 |
|  | Interview 1 | Knowledge about HIV | 18 | 18 | 0 | اینکه اگاهی پیدا کنی | bazrafshan | 16/11/2019 12:49 |  | 1 |
|  | Interview 1 | Communication support | 19 | 19 | 0 | عالی عالی الان دوستای خوبی دارم که 5 ساله باهاشون دوستم در ارتباطیم باهم خیلی منو دوست دارن بچه ها و به هم وابسته ایم از کوچک ترین مشکل تا بزرگترین مشکلشونو بچه ها تو اونجا به من عنوان میکنن میگن ... ما این مشکلو داریم الان این طوریم مجابشون میکنم که بیان تو کلاسا شرکت کنند مجابشون میکنم که بیان باهمدیگه در تعامل باشیم بخاطر این خیلی از بچه ها چه خانم چه اقا فرقی نمیکنه باهمشون در ارتباط | bazrafshan | 16/11/2019 12:50 |  | 1 |
|  | Interview 1 | Communication support\Peer communication | 19 | 19 | 0 | عالی عالی الان دوستای خوبی دارم که 5 ساله باهاشون دوستم در ارتباطیم باهم خیلی منو دوست دارن بچه ها و به هم وابسته ایم از کوچک ترین مشکل تا بزرگترین مشکلشونو بچه ها تو اونجا به من عنوان میکنن میگن ... ما این مشکلو داریم الان این طوریم مجابشون میکنم که بیان تو کلاسا شرکت کنند مجابشون میکنم که بیان باهمدیگه در تعامل باشیم بخاطر این خیلی از بچه ها چه خانم چه اقا فرقی نمیکنه باهمشون در ارتباطم | bazrafshan | 19/11/2019 11:43 |  | 1 |
|  | Interview 1 | Communication support | 20 | 20 | 0 | خاطر همین شبکه هارو بیشتر گذاشتیم که بچه ها باهم ارتباط تنگاتنگ داشته باشن بتونن مسائلشونو باهم درمیون بذارن در ارتباط باشن مسائلشونو مطرح کنن | bazrafshan | 16/11/2019 12:52 |  | 1 |
|  | Interview 1 | Communication support\Peer communication | 20 | 20 | 0 | بچه ها باهم ارتباط تنگاتنگ داشته باش | bazrafshan | 19/11/2019 11:44 |  | 1 |
|  | Interview 1 | Emotional support | 20 | 20 | 0 | احساس نزدیکی با همدیگه دارید؟ خیلی زیاد خیلی زیا | bazrafshan | 16/11/2019 12:50 |  | 1 |
|  | Interview 1 | Emotional support | 20 | 20 | 0 | همین شبکه هارو بیشتر گذاشتیم که بچه ها باهم ارتباط تنگاتنگ داشته باشن بتونن مسائلشونو باهم درمیون بذارن در ارتباط باشن مسائلشونو مطرح کنند | bazrafshan | 16/11/2019 12:51 |  | 1 |
|  | Interview 1 | Informational support | 20 | 20 | 0 | یماری ما جوریه که بعضی مسائل که برای بچه ها به وجود میاد نمیتونن توی جمع بگن مثلا یه خانم جوانی هست فوق العاده سنش پایینه ایشون زگیل تناسلی گرفته بود چندتا ناراحتی زنان گرفته بود مادرش توی پیج من امد گفت ... من اینو چکارش باید بکنم راهنماییش کردم بردم اینجا با دکتر صحبت کردیم بعد این بچه اصلن دارو نمیخورد مجابش کردیم با ترفندهایی که بتونم بکار ببرم مجابش کردیم که الان داره دارو میخوره..خیلی این شبکه ها اگر که ادامه داشته باشن اگر که ادم راحت باشه خیلی خوبه | bazrafshan | 16/11/2019 12:53 |  | 1 |
|  | Interview 1 | Access to information | 23 | 23 | 0 | وقتی ما مشکلی داریم دسترسی به دکتر نداریم یا تعطیله یا شبه یا بی موقعست یا مثلن دوستی اشنایی مثلن یکی از بچه میگه... ما این مشکلو داریم خودمون نمیتونیم با دکتر گفتمان کنیم تو تو پیجت بذار تو شما با ما بذار که ما این مشکلو داریم ببین عزیزم خیلی عالیه یعنی اصلن فکرش به ذهن ادم نمیاد شما الان من این برنامه شما با مارو نشون بدم میبینی در طول شبانه روز این فعاله یعنی فوق العادست یعنی که اگر این نباشه بچه ها خیلی از مشکلاتشون میمونه مثلن یکی از بچه ورامینه منزلش دسترسی به دکتر نداره یکی خوزستانه یکی اراکه یکی اهوازه یکی شیرازه یکی مشهده ...هرجای ایرون خب منتها این سراسریه جوریه که همه بچه ها میتونن ازش استفاده کنن بخاطر این من فکر میکنم خیلی عالیه من خودم به تنهایی مثلن اونو باز میکنم ببینم بچه ها چه سوالاتی کردند اطلاعات من بهش افزوده میشه دکتر جوابی که میده، چون من خودم همسانم استفاده می کنم | bazrafshan | 16/11/2019 12:54 |  | 1 |
|  | Interview 1 | Knowledge about HIV | 24 | 24 | 0 | والا ارتباط از زمانی که من اومدم اینجا هم برای ... اومدم هم برای ... از زمانی که اومدم خیلی چیزای جدید یاد گرفتم هم برای بیماریم از دکترای روانشناسی که هستند میان برامون گفتمان می کنند دکترای عفونی که هستند میان برامون گفتمان می کنند متخصص تغذیه داریم میان گفتمان می کنن علاوه بر اینکه در مورد بیماریمون خیلی چیزای جدید یاد میگیریم خیلی چیزام به اطلاعاتمون افزوده میش | bazrafshan | 16/11/2019 12:55 |  | 1 |
|  | Interview 1 | Sef efficacy & self-care behavior | 24 | 24 | 0 | ورزش میکنم پیاده روی میکنم شنا میرم بله استخر میرم مرتب تئاتر میرم سینما میرم کلن یک ادم استیبل و به روزم به بچه ها هم میگم اینجوری باشن | bazrafshan | 16/11/2019 13:22 |  | 1 |
|  | Interview 1 | Motivation & confidence | 26 | 26 | 0 | ه نظر من ارزش قائل بودن یک چیز عالیه میدونید چرا ؟چون وقتی تو این کارو میکنی به دوستت میفهمونی که تو به عنوان یک دوست به عنوان یک هم درد فوق العاده ای گوش میدم به صحبتاشون اگر هم اجرایی نکنم اونو بخاطر اینکه اون احساس کنه هدفمنده احساس کنه که ... دوسش داره انگیزه پیدا میکنه انگیزه پیدا کنه برای من قشنگه حس میکنم دوستم هم دردم از اینکه ... به حرفاش گوش میده مطالبشو نگاه میکنه ببینه چی براش فرستاده تاکید میکنم اگر خودم هم بیشتر ازون بدونم ولی باز هم استفاده میکنم دوست دارم اون برای من وقت گذاشته میدونی فکر کرده اگر این کارو کنه ... خوشحال میشه – بله چرا که نه | bazrafshan | 16/11/2019 12:57 |  | 1 |
|  | Interview 1 | Emotional support | 30 | 30 | 0 | باور کنید اگر که اینارو بردارند بچه ها خیلی اسیب می بینند چون میدونی مثلا با ی سیزده تومن میتونن بسته اینترنت بخرن حداقل جایی نمیتونن برن چت که میتونن بکنن صحبت که میتونن بکنن دلداری میتونن بکنن حالا حتمن که نباید بحث ارتباط مذکر و مونث با | bazrafshan | 16/11/2019 12:58 |  | 1 |
|  | Interview 1 | Emotional support | 32 | 32 | 0 | به همین دلیل فکر میکنم از 100 درصد 99 درصد این شبکه ها عالین علی الخصوص مرکز ما باشگاه ... پیج شما با ما چندتا پیج دیگه هم هست دوستان درست کردند خانم ... هست دوستان رو جمع میکنه جدیدترین و به روزترین چیزارو ارسال می کند صبح که پا میشی باز میکنی گوشی رو فوق العادست همه دوستا به هم پیام میدن روز بخیر میگن حالتون خوبه میگن از احوال همدیگه میپرسن جویای احوال همدیگه میشن خلاصه در کل گروه هغایی که مختص اینجا هست... بخش مشاورمون که پایین هست با دکترای متخصصمون بخش عفونی طبه ششم فکر کنم همشون عالی هستند بچه ها خیلی استقبال می کنند. | bazrafshan | 16/11/2019 13:00 |  | 1 |
|  | Interview 1 | Knowledge about HIV | 32 | 32 | 0 | یلی خوبه خیلی عالیه مثلا الان همین گروه شما و ما که داریم این فوق العاده است بهترین و جدیدترین و به روز ترین مطالب توش نوشته میشه که کجا چه اتفاقی برای بچه ها افتاده چه مسائلی دارن چه کار باید بکنن به روز باشن چه کمکی میتونن بکنن مثلا چه داروهایی استفاده بکنن چه داروهایی رو استفاده نکنن چه داروهایی براشون ضرر داره حتی این طب سنتی مثلا چیزای گیاهی بعضی ازین چیزای گیاهی رو نباید مصرف کنیم ، چیارو باید خورد چیارو نباید خورد کجاها بریم با کیا در ارتباط باشیم چه جوری برخورد کنیم اینا همش خیلی مهمه | bazrafshan | 16/11/2019 12:59 |  | 1 |
|  | Interview 1 | Disease perception | 34 | 34 | 0 | ادم از بوسیدن HIV میگیره از بزاق دهن بیماری میگیره از اب چشم ادم بیماری میگیره حتی استخر بری بیماری میگیری در صورتیکه این طور نیس | bazrafshan | 16/11/2019 13:22 |  | 1 |
|  | Interview 1 | Motivation & confidence | 34 | 34 | 0 | حرفهای ... مثلا این مشکلو داره ولی چه قشنگ استیبله داره زندگی میکنه ادم خیلی به روزیه انگیزه داره برای زندگ | bazrafshan | 16/11/2019 13:02 |  | 1 |
|  | Interview 1 | Recommendations for OSN improvements | 34 | 34 | 0 | یج هایی که مربوط به بیمارستانه شما باید پیج هایی رو بگذارید که این مسائل توش عنوان بشه مثلا منه ... بیام توش بصورت صوتی صحبت کنم اسم و فامیلم عنوان نشه ولی خیلی هارو مجاب کنم بشینن گوش بد | bazrafshan | 16/11/2019 13:01 |  | 1 |
|  | Interview 1 | Recommendations for OSN improvements | 34 | 34 | 0 | اگر شما یک پیجی رو درست کنید من ... قول میدم صوتی صحبت کنم جوونارو بخوام ازشون صحبت کنم بیام اینجا ازمایش بدن ازمایش مجانی | bazrafshan | 16/11/2019 13:04 |  | 1 |
|  | Interview 1 | Recommendations for OSN improvements | 34 | 34 | 0 | جازه نداریم مثلا اگه اجازه بدن من برم دبیرستان دخترانه صحبت کنم خیلی سخته باید اینجا نامه بگیریم خانم دکتر ... امضا کنه ببریم جاهای دیگه امضا کنند به سختی باید برم مدرسه با مدیرش صحبت کنم اون بهم بگه چی بگم چی نگم وقتی که من برم توی مدرسه ها صحبت کنم دختربچه هارو مجاب کنم بگم بچه هاراه انتقالش اینه برم مدرسه های پسرانه با بچه ها صحبت کنم بگم راه انتقالش اینه ...اگه شما یک پیج درست کنید تا این مسائل عنوان بشه خیلی خوب | bazrafshan | 16/11/2019 13:05 |  | 1 |
|  | Interview 1 | Disease perception | 35 | 35 | 0 | من خودم قبل از زمانی که به این بیماری مبتلا بشم وقتی از تلویزیوهن در مورد این بیماری میشنیدم میگفتم خدای من اگر من این بیماری رو بگیرم خواهم مرد یعنی اینقدر میترسیدم هراس داشتم چون اطلاع کافی نداشتم | bazrafshan | 16/11/2019 13:09 |  | 1 |
|  | Interview 1 | Communication support\trust | 41 | 41 | 0 | شبکه های مجازی خیلی از چیزاشون اصلا واقعیت نیست مثلا چند وقت پیش میگفتند که درمان HIV امده بچه ها خیلی ریختن به هم دارو نمیخوردند میگفتند داروش اومده ..قبول دارم باشه خوبه اما به نظر من برنامه اگر مختص اینجا باشه بهتره و مطئن تره ..چون خودشون در موردش تحصیل کردند سالها با مریضا در ارتباطند مثلا همین Imode که من زدم اطلاع داری | bazrafshan | 16/11/2019 13:11 |  | 1 |
|  | Interview 1 | Emotional support | 45 | 45 | 0 | .بچه ها وقتی باهم در تعاملن حالشون خوب میش | bazrafshan | 16/11/2019 13:13 |  | 1 |
|  | Interview 1 | Communication support\trust | 47 | 47 | 0 | ثل همون مورد که گفتند درمانش اومده منکه باور نمیکنم این حرفارو چه بسا که اگر اومده باشه اینجا بهمون میگفتند اینارو وقتی تو شبکه ها مینویسند باورش برای بچه ها خیلی سخته ولی وقتی بچه ها میان اینجا ..میگن واقعیت داره یا نه ..من میگم هر دو مکمل هم اند | bazrafshan | 16/11/2019 13:14 |  | 1 |
|  | Interview 1 | Knowledge about HIV | 47 | 47 | 0 | خیلی ..خیلی زیاد 70 درصد این طورا..ا | bazrafshan | 16/11/2019 13:13 |  | 1 |
|  | Interview 1 | Access to information | 49 | 49 | 0 | خیلی از این ادمها نمیتونن بیان تو جلسات خیلی از بچه ها برای درمان نمیان اینقد ازرده خاطر شدن مشکل دارن زندگیشون بهم ریخته خیلی از بچه ها بیمارن هنوز حالشون بد شده زخم میگیرن هپاتیت دارن نمیان اینجورجاها شبکه های اجتماعی برای اونا خوبه حالشون خوب میشه اونو حداقل باز میکنن یه سرچ میکنن ببینن چه خبره شاد حال خوب بهشون بد | bazrafshan | 16/11/2019 13:14 |  | 1 |
|  | Interview 1 | Communication support | 52 | 52 | 0 | اگر که هماهنگ باشه با پزشکان حازق این کار خیلی عالیه اگر همچین اتفاقی بیوفته من خودم که زیاد تمایلی به شبکه های اجتماعی ندارم استقبال میکنم و دوست دارم | bazrafshan | 16/11/2019 13:15 |  | 1 |
|  | Interview 1 | Communication support\trust | 63 | 63 | 0 | ک متولی رسمی داشته باشه تا به اطلاعات ان بشه اعتماد کرد | bazrafshan | 16/11/2019 13:16 |  | 1 |
|  | Interview 1 | Recommendations for OSN improvements | 63 | 63 | 0 | وزارت بهداشت خودش پیجی رو درست کنه ادمهایی که توی این زمینه تحصیل کردند اونجا باشند و به بچه مشاره بدهند این خیلی عالی و وفوق العادست در کنارش خدمات این مرکز هم باش | bazrafshan | 16/11/2019 13:16 |  | 1 |
|  | Interview 1 | Adherence | 66 | 67 | 0 | کنید حضور در شبکه های اجتماعی چقدر بقیه را به ادامه درمان ترغیب می کند؟ 99 درص | bazrafshan | 16/11/2019 13:20 |  | 1 |
|  | Interview 1 | Emotional support | 67 | 67 | 0 | چی با گفتمان عالیه وقتی یکی حال بدی داره با شما صحبت میکنه صادقانه به حرفاش گوش میدی اون حس میکنه یکی هست حامیش باش | bazrafshan | 16/11/2019 13:17 |  | 1 |
|  | Interview 1 | Motivation & confidence | 67 | 67 | 0 | 99 درص | bazrafshan | 16/11/2019 13:17 |  | 1 |
|  | Interview 1 | Communication support\trust | 70 | 70 | 0 | گر زیر مجموعه وزارت باشه پرسنل اموزش دیده باشن حرفاشون برپایه واقعیته بچه هاو الکی تحریک نمیکنن و اطلاعات صحیح بدن خوب | bazrafshan | 16/11/2019 13:18 |  | 1 |
|  | Interview 1 | Disease perception | 72 | 72 | 0 | الاعات خیلی مهمه که چه اتفاقاتی براش میوفته پیرفتن مردم و جامعه ..اگه کسی بفهمه من بیمارم با من ازدواج نمیکنه از من دور میشن من بمیرم بهتره ...اگر بشه حمایشتون کرد وقتی اجازه داشته باشی پیش همه مردم بری و عنوان کنی قبح قضیه برطرف بشه بفهمن که این بیماری مهلک نیست ..میتونیم ازدواج کنیم با فرزندانمون زندگی کنیم ولی وقتی مردم اطلاعات کافی نداشته باشن تاثیر میگذاره وقتی من قراره بمیرم هیشکی منو قبول نداره ...منم نمیام اینج | bazrafshan | 16/11/2019 13:18 |  | 1 |
