# Supplementary material for: The role of online social networks in improving health literacy and medication adherence among people living with HIV/AIDS in Iran: Development of a conceptual model
Source: PLoS One. 2022 Jun 30;17(6):e0261304. doi: 10.1371/journal.pone.0261304 (PMC9246123; doi:10.1371/journal.pone.0261304)
Supplement: S2 File — (PDF) [file pone.0261304.s004.pdf]

Code System [948]
- **anonymous identity [3]**
- **age [28]**
- **education [27]**
- **history of drug abuse [25]**
- **employment status [28]**
- **date of diagnosis [29]**
- **Sex [0]**
  - **male [18]**
  - **female [11]**
- **way of transmission [28]**
- **Disease perception [16]**
- **Knowledge about HIV [45]**
- **Access to information [16]**
- **Emotional support [53]**
- **Informational support [67]**
- **Negative emotions [2]**
- **Instrumental support [10]**
- **Barriers [58]**
- **OSN app [39]**
- **OSN group [31]**
- **Recommendations for OSN improvements [45]**
- **Time of using OSN [25]**
- **Overall impression [70]**
- **Sef efficacy & self-care behavior [19]**
- **Adherence [33]**
- **Motivation & confidence [56]**
- **OSN outcome [0]**
- **Overall OSN Use & Impression [2]**
- **Communication support [53]**
  - **Peer communication [33]**
  - **Patient-provider [26]**
  - **trust [52]**
- **Social support [0]**
- **Demographic information [0]**

Sets [0]
