# Supplementary material for: The role of online social networks in improving health literacy and medication adherence among people living with HIV/AIDS in Iran: Development of a conceptual model
Source: PLoS One. 2022 Jun 30;17(6):e0261304. doi: 10.1371/journal.pone.0261304 (PMC9246123; doi:10.1371/journal.pone.0261304)
Supplement: Attachment — Submitted filename: PONE-D-21-04415-The role of online social networks.docx [file pone.0261304.s005.docx]

**Reviewer’s Comments**

**05 May 2021**

***The Editor-In-Chief***

***PONE***

**Re: PONE-D- 21-04415: The role of online social networks in improving health literacy and medication adherence among people living with HIV/AIDS in Iran: A mixed-method study**

Thank you for the opportunity to review the above-named manuscript. The investigators have conducted an important study primarily aimed to develop a conceptual model of using online social networks in improving health literacy and medication adherence among people living with HIV/AIDS in Iran. The study is not novel as several previous studies were conducted from other places. However, to my knowledge, this is the first from Iran. If well-conducted and reported, the study will add important additional information to the existing body of literature related to online social networks in improving health literacy and medication adherence in PLWHA. I have made recommendations for improvement of the manuscript.

**General**

- The manuscript is generally well-written. However, it contains a few grammatical errors and need for syntax improvement. The paper will benefit from careful proof-reading before it will be considered for publication in the journal.
- My major concern with the study is that it is unclear if the Delphi and the nominal group technique were robust enough to yield a valid conceptual model. More information may be required.
- Another concern is that there is no sufficient information on the items that were used in the questionnaire utilized in the Delphi phase.
- As a normal scientific writing convention, abbreviations should always be defined in full-term at first use and the abbreviation used thereafter.

**Title**

- I am not sure, but I doubt if the study can be described as a “mixed-methods”. In the secondary title, can it be described as “a multi-methods study” or simply “development of a conceptual model”?

**Abstract**

- The abstract is well-written.
- Abbreviations such as HIV should be defined in full-term at first use.
- While the study is described as three phases, I do not seem to see the third phase in the abstract. I understand the qualitative part as one phase and Delphi as another phase. What is the third phase?
- Line 32: Change “24 sub-themes were emerged” to “24 sub-themes emerged”.

**Introduction**

- The Introduction is fairly well-written, and has provided some insight about what is known in the literature and the gaps, but it can be better.
- However, some redundancies and repetitions regarding the use and value of Internet and OSN among PLWHA. This can be better by minimizing the redundancies.
- As a normal scientific writing convention, abbreviations should always be defined in full-term at first use and the abbreviation used thereafter. Examples HIV/AIDS, ART, PLWHA etc.
- Line 58-61: Any references to support the statements in line 58 - 61?
- Line 62: The open bracket before OSN should be closed.
- Line 74: A cross-sectional” instead of “Across”.
- Line 81: “patient-cantered” should be “patient-centered”
- Aim of the study: The aim in the Introduction is different from the one in the Abstract. The researchers need to be consistent.

**Methods**

- Line 98-99: Summarize the three sequential phases and justify the choice of this approach. It also must be well clear what you aim to achive in phase 2 and phase 3 and how they are connected.
- Line 122: You said interviews duration ranged between 14 - 60 minutes and the longest lasted for 90 minutes. This is a contradiction.
- Line 123: Change to “… were conducted”.
- Phase 2 and Phase 3: The process for the development of conceptual model is rather superficial and not detailed enough. More information and robust process are needed.
- Phase 2 (Delphi study): It is unclear how many rounds of Delphi were conducted.
- Phase 3 (Nominal group technique): It is unclear how the NGT differs from the Delphi in terms of methodology. I am also not clear how many members were included in total. If n=5, is this considered adequate?
- Line 141: Was online social networks not abbreviated as OSN before?
- Line 148 and throughout the paper: British vs. US English.

**Results**

- Generally well-written with clear tables to support the textual descriptions.
- Line 171 – 183: Demographic information should have also been presented in a table.
- Line 182: Thirteen of 29 does not represent 51%.
- Line 192: Change “retroviral” to “anti-retroviral”
- Line 192: Are you sure there are four sub-themes? I counted 5 in Table 1.
- Line 411: In the Delphi methods, the level of agreement was 75%. Here you have changed to 70%. Be consistent.

**Discussion**

- The Discussion needs to be more critical and supported by relevant literature and not merely a summary of the results.
- The discussions are redundant in places. These must focus on the major findings and citing relevant literature. Several important previous studies not cited.

**Conclusion**

- Can be better written to reflect the study objectives and finding. In addition, how would the conceptual model help in designing interventions to improve medication literacy and medication adherence through OSN?

**References**

- I advise that the authors should strictly adhere to the journal’s referencing style (please see authors instructions).
- Some references are incomplete and inaccurate.
- In addition, some journals names are not according to the journal’s style.
