# Supplementary material for: The role of online social networks in improving health literacy and medication adherence among people living with HIV/AIDS in Iran: Development of a conceptual model
Source: PLoS One. 2022 Jun 30;17(6):e0261304. doi: 10.1371/journal.pone.0261304 (PMC9246123; doi:10.1371/journal.pone.0261304)
Supplement: Attachment — Submitted filename: Response to reviewers.docx [file pone.0261304.s006.docx]

**Response to reviewers**

**07 May 2021**

***Dear Editor,***

**Re: PONE-D-21-04415: The role of online social networks in improving health literacy and medication adherence among people living with HIV/AIDS in Iran: Development of a conceptual model**

Thank you for giving us the opportunity to submit a revised draft of the manuscript for publication in the Journal of PLOS ONE. We appreciate the time and effort that you and the reviewers dedicated to providing feedback on our manuscript and are grateful for the insightful comments on and valuable improvements to our paper. We have incorporated most of the suggestions made by the reviewers. Those changes are highlighted within the manuscript. Please see below, in blue, for a point-by-point response to the reviewers’ comments and concerns. All page numbers refer to the revised manuscript file with tracked changes.

**Reviewer comments to the authors**

**General**

- The manuscript is generally well-written. However, it contains a few grammatical errors and need for syntax improvement. The paper will benefit from careful proof-reading before it will be considered for publication in the journal.

Thank you! We have revised the grammar and writing of the manuscript again to improve the language and the quality of writing.

- My major concern with the study is that it is unclear if the Delphi and the nominal group technique were robust enough to yield a valid conceptual model. More information may be required.

Thank you. We agree with the reviewer. Further information about the technical aspects of the Delphi study and the nominal group technique were added to the method’s section.

- Another concern is that there is no sufficient information on the items that were used in the questionnaire utilized in the Delphi phase.

Thank you. We agree with the reviewer. Further information about designing the questionnaire and survey items used in the Delphi study were added to the method’s section.

- As a normal scientific writing convention, abbreviations should always be defined in full-term at first use and the abbreviation used thereafter.

We agree with the reviewer’s assessment. Accordingly, throughout the manuscript, we have revised the abbreviations and corrected the typos.

**Title**

- I am not sure, but I doubt if the study can be described as a “mixed-methods”. In the secondary title, can it be described as “a multi-methods study” or simply “development of a conceptual model”?

The title was edited as you pointed out.

**Abstract**

- The abstract is well-written.
- Abbreviations such as HIV should be defined in full-term at first use.

We agree with the reviewer’s assessment. Accordingly, throughout the manuscript, we have revised the abbreviations and corrected the typos

- While the study is described as three phases, I do not seem to see the third phase in the abstract. I understand the qualitative part as one phase and Delphi as another phase. What is the third phase?

We have considered the nominal group technique as the third phase of the study.

- Line 32: Change “24 sub-themes were emerged” to “24 sub-themes emerged”.

Modifications were made as you pointed out.

**Introduction**

- The Introduction is fairly well-written, and has provided some insight about what is known in the literature and the gaps, but it can be better.

Some explanations were added to the introduction section as you pointed out.

- However, some redundancies and repetitions regarding the use and value of Internet and OSN among PLWHA. This can be better by minimizing the redundancies.

Modifications were made as you pointed out.

- As a normal scientific writing convention, abbreviations should always be defined in full-term at first use and the abbreviation used thereafter. Examples HIV/AIDS, ART, PLWHA etc.

We agree with the reviewer’s assessment. Accordingly, throughout the manuscript, we have revised the abbreviations and corrected the typos

- Line 58-61: Any references to support the statements in line 58 - 61?

New references were added to the manuscript.

- Line 62: The open bracket before OSN should be closed.

Modifications were made as you pointed out.

- Line 74: A cross-sectional” instead of “Across”.

Modifications were made as you pointed out.

- Line 81: “patient-cantered” should be “patient-centered”

Modifications were made as you pointed out.

- Aim of the study: The aim in the Introduction is different from the one in the Abstract. The researchers need to be consistent.

Modifications were made as you pointed out.

**Methods**

- Line 98-99: Summarize the three sequential phases and justify the choice of this approach. It also must be well clear what you aim to achieve in phase 2 and phase 3 and how they are connected.

Some explanations were added to the method section as you pointed out.

- Line 122: You said interviews duration ranged between 14 - 60 minutes and the longest lasted for 90 minutes. This is a contradiction.

Modifications were made as you pointed out.

- Line 123: Change to “… were conducted”.

Modifications were made as you pointed out.

Phase 2 and Phase 3: The process for the development of conceptual model is rather superficial and not detailed enough. More information and robust process are needed. Some explanations about the techniqual aspects of the Delphi study and development of the conceptual model were added as you pointed out.

- Phase 2 (Delphi study): It is unclear how many rounds of Delphi were conducted.
- Modifications were made as you pointed out.
- Phase 3 (Nominal group technique): It is unclear how the NGT differs from the Delphi in terms of methodology. I am also not clear how many members were included in total. If n=5, is this considered adequate?
- Modifications were made as you pointed out.
- Line 141: Was online social networks not abbreviated as OSN before?
- Modifications were made as you pointed out.
- Line 148 and throughout the paper: British vs. US English.
- Modifications were made as you pointed out.

**Results**

- Generally well-written with clear tables to support the textual descriptions.

Thank you!

- Line 171 – 183: Demographic information should have also been presented in a table. A new table was inserted to the manuscript to summarize the demographic data of respondents.
- Line 182: Thirteen of 29 does not represent 51%.
- Modifications were made as you pointed out
- Line 192: Change “retroviral” to “anti-retroviral”
- Modifications were made as you pointed out
- Line 192: Are you sure there are four sub-themes? I counted 5 in Table 1.
- Modifications were made as you pointed out
- Line 411: In the Delphi methods, the level of agreement was 75%. Here you have changed to 70%. Be consistent.

Modifications were made as you pointed out

**Discussion**

- The Discussion needs to be more critical and supported by relevant literature and not merely a summary of the results.
- The discussions are redundant in places. These must focus on the major findings and citing relevant literature. Several important previous studies not cited.

Thank you! We have modified the discussion and classified the reference to a new format as you pointed out.

**Conclusion**

- Can be better written to reflect the study objectives and finding. In addition, how would the conceptual model help in designing interventions to improve medication literacy and medication adherence through OSN?

Conclusion was revised and implications of the proposed model were added.

**References**

- I advise that the authors should strictly adhere to the journal’s referencing style (please see authors instructions).
- Some references are incomplete and inaccurate.
- In addition, some journals names are not according to the journal’s style.

Thank you! All journal names were checked by the NLM journal database and the abbreviations were added. Reference styles were again checked by the Vancouver style.
