# Supplementary material for: The role of online social networks in improving health literacy and medication adherence among people living with HIV/AIDS in Iran: Development of a conceptual model
Source: PLoS One. 2022 Jun 30;17(6):e0261304. doi: 10.1371/journal.pone.0261304 (PMC9246123; doi:10.1371/journal.pone.0261304)
Supplement: Attachment — Submitted filename: Rebuttal letter.docx [file pone.0261304.s007.docx]

Dear reviewers,

Thank you so much for all your insightful comments. Our manuscript’s language has been recently evaluated by an expert English Language researcher and we have made some modifications to improve the quality of the grammar and writing style of the manuscript. We have also checked the reference style. In addition, we have tried to improve the format of the qualitative results in Table 2. The co-authors have checked the open codes and sub-themes carefully. But they have decided not to change the current format. We really appreciate your dedication and commitment to improve the quality of this manuscript. Hope these modifications comply with your remarks.

Sincerely yours,

Sirous Panahi

Corresponding author
